# Clinicopathologic Characteristics, Molecular Profile, Treatment, and Recurrence in Endometrial Cancer with Pelvic Lymph Node Isolated Tumor Cells

**DOI:** 10.3390/cancers18203293

**Published:** 2026-10-13

**Authors:** Haley D. Frerichs, Cheng-Han Lee, Ericka Wiebe, Sophia Pin

**Affiliations:** 1 Faculty of Medicine & Dentistry, University of Alberta, Edmonton, AB T6G 2R3, Canada; 2 Department of Laboratory Medicine and Pathology, University of Alberta, Edmonton, AB T6G 2R3, Canada; cheng-han.lee@albertaprecisionlabs.ca; 3 Cross Cancer Institute, Department of Oncology, Faculty of Medicine & Dentistry, University of Alberta, Edmonton, AB T6G 2R3, Canada; 4 Cross Cancer Institute, Department of Obstetrics & Gynecology, Division of Gynecologic Oncology, Faculty of Medicine & Dentistry, University of Alberta, Edmonton, AB T6G 2R3, Canada

**Keywords:** endometrial cancer, isolated tumor cells, sentinel lymph node biopsy

## Abstract

Objective: Our objective was to characterize the molecular–pathologic profile and recurrence patterns of endometrial cancer with pelvic lymph node isolated tumor cells (ITCs). Methods: A single-center retrospective study of patients with endometrial cancer and ITCs was conducted. Patients with concurrent lymph nodes or distant metastasis were excluded. Cases were reviewed by a gynecologic pathologist, and electronic medical records were reviewed. Descriptive statistics were performed using R 4.4.2. Results: A total of 43 patients were included, with a median follow-up time of 33 months. The majority were endometrioid carcinoma (91%) and stage I (72%). In total, 28 (65%) were MMR-proficient and p53-wild-type (MMRp p53wt), nine (21%) were MMR-deficient (MMRd), six (14%) were p53-abnormal (p53abn), and none were *POLE*-mutated (*POLE*mut). Additionally, 27 (96%) MMRp p53wt cases showed a microcystic, elongated, and fragmented (MELF) pattern of invasion. Adjuvant therapy was indicated in 38 (88%) patients; 32 (74%) received therapy and six (14%) declined. Four (9.3%) patients recurred, with a recurrence rate of 33% (2/6) for p53abn, 11% (1/9) for MMRd, and 4% (1/28) for MMRp p53wt. All recurrences were to distant sites. In the MMRp p53wt group, 16 (57%) had external beam radiation (EBRT) +/− vaginal brachytherapy (VBT), three (11%) had chemotherapy +/− radiation, and nine (32%) had no adjuvant treatment or VBT alone. Conclusions: Molecular profile is prognostically relevant in endometrial carcinoma with ITCs. ITCs in MMRp p53wt endometrioid carcinoma present a low risk of disease recurrence. As the majority of our cohort received adjuvant therapy, future work may explore de-escalating treatment in low-risk ITC cases.

## Simple Summary

Isolated tumor cells (ITCs) are small groups of cancer cells found in lymph nodes with uncertain significance in endometrial cancer. We retrospectively reviewed 43 patients with endometrial cancer and pelvic lymph node ITCs and examined their clinicopathological features, molecular subtype, treatment, and recurrence patterns. Most patients had endometrioid tumors, and MMRp p53wt tumors were the most common molecular subtype. Four patients (9%) experienced recurrence during follow-up, including one patient with MMRp p53wt disease, one with MMRd disease, and two with p53abn disease. Most patients received adjuvant treatment, particularly external beam radiation therapy, so the observed recurrence rates cannot be separated from the effects of treatment. Larger studies with complete molecular classification, longer follow-up, and appropriate ITC-negative comparison groups are needed.

## 1. Introduction

Endometrial cancer remains the most common gynecologic malignancy in North America. While advances in both detection and treatment have led to high overall survival rates, the incidence of endometrial cancer is increasing worldwide, and a subset of patients still experience recurrence despite treatment [1]. This highlights the need for more tailored approaches to endometrial cancer care, with molecular profiling increasingly being used to guide treatment decisions. The four main endometrial cancer molecular subtypes, as first determined by The Cancer Genome Atlas (TCGA), are p53-abnormal (p53abn), mismatch-repair-deficient (MMRd), *POLE*-mutated (*POLE*mut), and non-specific molecular profile (NSMP) [2]. Given its poor prognosis, p53abn endometrial cancer is typically treated with more aggressive adjuvant therapies, usually involving chemotherapy with or without radiation. In contrast, adjuvant treatment is frequently de-escalated in *POLE*mut endometrial cancer due to its excellent prognosis [3]. Outcomes for MMRd and NSMP endometrial cancers are intermediate, highlighting the need for further refinement of treatment strategies tailored to these molecular subtypes [2].

The standard of care for presumed early-stage endometrial cancer is total hysterectomy with bilateral salpingo-oophorectomy and pelvic sentinel lymph node biopsy for staging [3]. The use of sentinel lymph node sampling with more comprehensive pathologic evaluation via ultrastaging has led to an increased recognition of isolated tumor cells (ITCs), defined as clusters of cancer cells measuring ≤0.2 mm in diameter or consisting of fewer than 200 cells [4,5]. ITCs are distinct from micrometastases, which range in size from >0.2 mm to 2 mm, and macrometastases, which exceed 2 mm in size. Unlike these larger nodal metastases, which are staged according to FIGO 2023 as IIIC1i (micrometastases) and IIIC1ii (macrometastases), the presence of ITCs does not result in the upstaging of endometrial cancer [6]. Further, there are currently no established treatment guidelines for endometrial cancer with pelvic lymph node ITCs, nor is it clear whether ITCs represent a risk factor for recurrence, especially in otherwise low-risk endometrial cancers.

The primary objective of this study was to describe recurrence patterns among patients with endometrial cancer and pelvic lymph node ITCs according to their available molecular classification, with particular attention to the MMR-proficient p53-wild-type (MMRp p53wt) subgroup, as this category requires prognostic refinement. The secondary endpoints included clinicopathological characteristics and adjuvant treatment patterns among patients with pelvic lymph node ITCs. Exploratory endpoints included associations between clinicopathological features, including lymphovascular space invasion (LVSI) and microcystic, elongated, and fragmented (MELF) pattern of invasion, molecular classification, and recurrence.

## 2. Materials and Methods

### 2.1. Study Cohort

This was a retrospective, single-institution observational cohort study of endometrial cancer patients with pelvic lymph node ITCs. Institutional Review Board approval was obtained prior to beginning the study (HREBA.CC-24-0233).

Consecutive patients with ITCs were identified using a prospectively collected database of all patients with endometrial cancer who underwent robotic hysterectomy with bilateral salpingo-oophorectomy at our institution from July 2015, when our institution began recognizing pelvic lymph node ITCs, to May 2024. Additional patients with ITCs not yet added to the database were identified by reviewing documentation from weekly gynecologic oncology tumor boards from January 2023 to October 2024.

Patients were included if they had pathologically confirmed stage I-III endometrial cancer, underwent hysterectomy and bilateral salpingo-oophorectomy with a pelvic sentinel lymph node biopsy or lymphadenectomy, and had pathology-confirmed pelvic lymph node ITCs. Patients with concurrent lymph node micrometastases or macrometastases or those with synchronous primary cancers were excluded. Patients who did not have at least one pathologically confirmed lymph node resected were excluded. All cases were reviewed by a gynecologic pathologist.

### 2.2. Treatment

All patients were referred to a gynecologic oncologist for the treatment of endometrial cancer or atypical endometrial hyperplasia. Patients with presumed early-stage disease (no sign of local or distant metastasis) underwent hysterectomy with bilateral salpingo-oophorectomy. The surgical procedure was usually done via robot-assisted laparoscopy, with laparotomy done in select cases when a minimally invasive approach was not feasible. All patients had a lymph node procedure, either a sentinel lymph node biopsy or a lymphadenectomy. Most underwent sentinel lymph node biopsy with indocyanine green (ICG), although methylene blue was used in some cases if ICG was unavailable.

All cases with pelvic lymph node ITCs were reviewed at multidisciplinary tumor board meetings to provide treatment recommendations. Patients who received adjuvant chemotherapy typically received six cycles of carboplatin and paclitaxel chemotherapy. When adjuvant external beam radiation therapy (EBRT) was recommended, the typical regimen consisted of 4500 cGy in 25 fractions. Patients who received adjuvant vaginal brachytherapy (VBT) typically received 2100 cGy over three treatments, or one treatment of 700 cGy if given in combination with EBRT. No patients received neoadjuvant treatment.

### 2.3. Pathology Evaluation

All cases were reviewed by a single gynecologic pathologist. Pelvic lymph node ITCs were defined according to the FIGO definition as tumor deposits measuring ≤0.2 mm or consisting of fewer than 200 tumor cells [6]. The MELF pattern of myometrial invasion was assessed according to the criteria described in the original publication by Murray et al. [7]. LVSI was assessed using a combination of histologic morphology and immunohistochemistry. LVSI was considered present when tumor emboli were identified within endothelial-lined vascular or lymphatic spaces, with CD31 and ERG immunohistochemistry used to support the identification of endothelial-lined spaces where applicable [8]. LVSI was described as absent, focal or diffuse, as outlined by Restaino et al. [9]. In cases where focal versus diffuse LVSI was not specified, LVSI was described as present.

For cases submitted with sentinel node(s), all nodes were thinly sectioned and submitted in toto for histologic evaluation, which involved keratin immunohistochemistry (IHC) analysis if no histologically apparent nodal metastases were identified [10]. For non-sentinel nodes, all nodes were submitted in toto for histologic evaluation as well, but keratin immunohistochemistry analysis was performed at the discretion of the case pathologists.

Molecular profile was assigned based on the four molecular subtypes defined by TCGA [2]. Patients were defined as MMRd if there was a loss of MMR proteins on IHC. Patients whose tumors were MMR-proficient (MMRp) with aberrant p53 IHC staining were classified as p53abn. Patients with MMRp tumors and wild-type p53 IHC staining (p53wt) were classified as MMRp p53wt. Because *POLE* testing was not available for all patients, MMRp p53wt tumors were not considered definitively classified as NSMP.

### 2.4. Chart Review for Treatment and Follow-Up Data

A chart review was conducted for the included patients using electronic medical records. Data extracted included demographics, pre- and post-operative diagnosis, details of adjuvant therapy, recurrence details, and survival.

### 2.5. Statistical Analysis

Data were summarized using descriptive statistics. Frequencies and percentages were calculated for categorical variables, and mean and standard deviation or median and IQR were calculated for continuous variables.

The estimated prevalence of pelvic lymph node ITCs in patients at our center was calculated only using patients identified from the database, not from tumor board records, to reduce selection bias.

Overall follow-up duration was calculated from the date of surgery to the date of the most recent follow-up or death, regardless of recurrence status, up until June 2025. Overall survival was defined as the time from surgery to death from any cause, with patients alive at last follow-up censored at that date. Time to recurrence was calculated from the date of surgery.

Statistical analyses were performed using R 4.4.2.

### 2.6. Narrative Literature Review

A literature search was performed to review adjuvant treatment decisions and recurrence patterns of endometrial cancer with ITCs. A search of MEDLINE from 1946 to 4 April 2025 was performed using the terms “endometrial cancer,” “isolated tumor cells,” and “recurrence.”

The inclusion criteria were endometrial cancer with pelvic lymph node ITCs, defined as a diameter of less than or equal to 0.2 mm or less than 200 cells, and details of recurrence. Studies that pooled pelvic lymph node micrometastases or macrometastases with ITCs were excluded.

Seventy-four studies were identified by the MEDLINE search, and an abstract screen then followed. After the abstract screen, 23 full-text articles were reviewed, and nine were excluded. Fourteen studies met the inclusion criteria [7,8,9,10,11,12,13,14,15,16,17,18,19,20].

## 3. Results

### 3.1. Demographics

**Figure 1:**
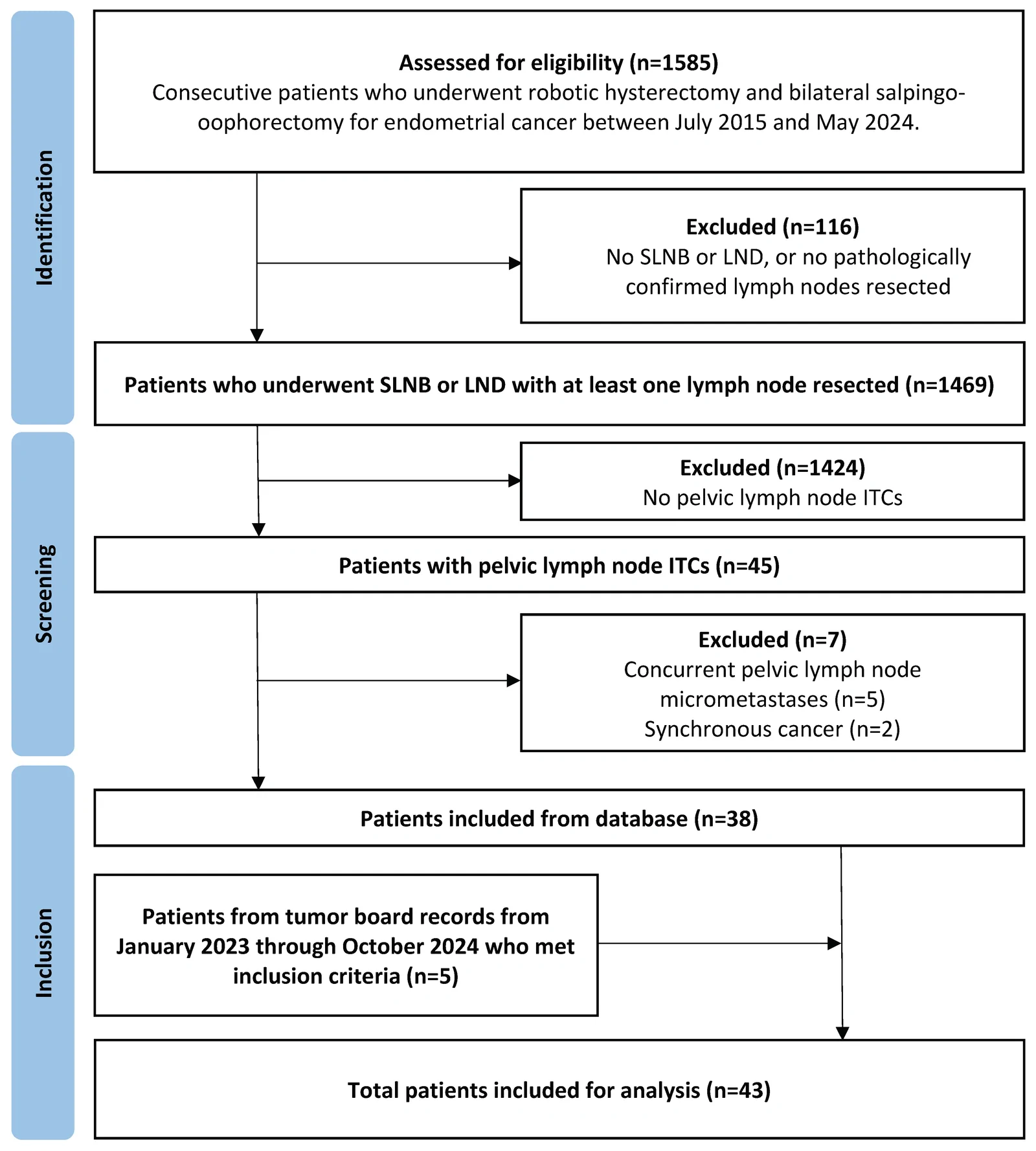
Flow chart of patients through the study. SLNB, sentinel lymph node biopsy; LND, lymphadenectomy; ITCs, isolated tumor cells.

Forty-three patients with stage I-III endometrial cancer and pelvic lymph node ITCs were included in the study. The flow of patients through the study is illustrated in Figure 1. Patients had a mean age of 66 years (SD = 10.6), with a mean BMI of 35 (SD = 8). The median follow-up time was 33 months (IQR = 16–60 months).

Of 1469 consecutive patients who underwent robotic hysterectomy, bilateral salpingo-oophorectomy, and sentinel lymph node biopsy or lymphadenectomy for endometrial cancer at our center, 45 patients had pathologically confirmed pelvic lymph node ITCs, making the prevalence of ITCs as the only evidence of nodal involvement and extrauterine spread at our center 3%.

Regarding surgical treatment, 40 (93%) received robot-assisted surgery, one (2%) initially received robotic-assisted surgery but was converted to laparotomy, and two (5%) were planned laparotomies. Thirty-eight (88%) had a sentinel lymph node biopsy with or without lymphadenectomy, while five (12%) had lymphadenectomy alone. All but three sentinel lymph node procedures were bilateral. A median of three pelvic lymph nodes were sampled (IQR 2–5) per patient.

### 3.2. Pathologic Characteristics

The pathologic features are summarized in Table 1. The majority of endometrial carcinomas were of endometrioid histology (91%), grade 1 (56%), and pathologic stage I (72%). Thirty-five (81%) demonstrated a MELF pattern of invasion. All patients underwent p53 and MMR testing. Thirteen (30%) patients had *POLE* testing performed, and all were negative for *POLE* mutations. Nine (21%) patients were MMRd, six (14%) were p53abn, and 28 (65%) were MMRp p53wt. Of the 28 MMRp p53wt patients, nine (32%) had *POLE* testing, with no mutations identified.

Regarding ITCs, 36 (84%) patients had ITCs identified in one pelvic lymph node, and seven (16%) had ITCs in more than one lymph node (range 2–5 lymph nodes). Six (86%) of the patients with multiple ITC-positive nodes were in the MMRp p53wt group, and one (14%) was in the MMRd group. All patients in the p53abn group had a single lymph node with ITCs identified.

Pathologic characteristics of the 28 patients with MMRp p53wt molecular profile are shown in Table 2. All patients in this molecular group were of endometrioid histology, and all but one (96%) had a MELF pattern of invasion. The majority were FIGO grade 1 (68%) and stage IB (54%). Four were stage IA (14%). All but seven (75%) had LVSI, with 10 (36%) demonstrating diffuse LVSI.

### 3.3. Clinical Management

An overview of adjuvant treatment decisions for patients with endometrial cancer and ITCs is provided in Table 3. Thirty-two of 43 (74%) patients with ITCs received adjuvant therapy. Of the 11 (26%) who did not receive adjuvant therapy, it was contraindicated in one (9%) patient, declined by six (55%) patients, and not indicated for four (36%) patients.

In total, 24 (56%) patients received adjuvant EBRT. This was combined with VBT in five patients (12%), with chemotherapy in two (5%) patients and with both chemotherapy and VBT in three (7%) patients. Two (8%) patients did not complete the full recommended course of EBRT (1260 cGy instead of the recommended 4500 cGy). Nineteen (79%) of 24 patients received 4500 cGy of EBRT, though dosing ranged from 1620 to 5750 cGy. EBRT was almost always administered in 25 fractions, though one (4%) patient received 23 fractions, and the two (8%) patients who completed a partial course received nine fractions.

Eight (19%) patients received chemotherapy, which consisted of carboplatin and paclitaxel in six (75%) and carboplatin alone in two (25%) patients. Most patients completed six cycles (75%), except one (12.5%) who completed five cycles and one (12.5%) who completed four cycles. Two (5%) patients received chemotherapy alone, and it was combined with VBT in one (2%) patient. The remaining five (12%) were combined with EBRT as outlined above.

Fourteen (33%) patients in total received VBT, with five (12%) receiving brachytherapy alone. Six (43%) patients received 2100 cGy in three fractions, seven (50%) received 700 cGy in one fraction, and one (7%) received 1500 cGy in three fractions.

In patients with the MMRp p53wt molecular profile, 18 (64%) received adjuvant pelvic EBRT. This was combined with VBT in four (14%) patients and with both chemotherapy and VBT in two (7%) patients. One (4%) patient received chemotherapy alone, and three (11%) received VBT alone. Six (21%) patients did not receive adjuvant therapy. Adjuvant therapy was recommended in three of these six patients; however, they declined treatment.

In the p53abn group, all received adjuvant treatment, with five of six (83%) receiving chemotherapy. This was combined with EBRT in two (33%) patients and with both EBRT and VBT in one (17%) patient. One (17%) patient received VBT alone. Of note, this patient had a mixed endometrial carcinoma with the serous p53abn component confined to a polyp, and the remainder of the cancer being endometrioid MMRp p53wt.

In the MMRd group, two (22%) patients received EBRT alone, one (11%) received VBT alone, and one (11%) received EBRT with VBT. Five (56%) patients did not receive adjuvant treatment. One patient declined, and one did not undergo adjuvant treatment due to significant medical comorbidities. Adjuvant treatment was not indicated in the remaining three patients. Of note, two patients in this group had unknown MMR status at the time of treatment and were retrospectively found to be MMRd. However, both these patients had stage IA endometrioid endometrial carcinoma without LVSI, and neither was offered adjuvant therapy.

### 3.4. Clinical Outcomes

Overall, four out of 43 (9%) patients had a recurrence over a median follow-up time of 33 months (IQR 14–54 months). The median time to recurrence was 13 months (IQR 8–31 months). The rates of recurrence were 4% (1 of 28) in the MMRp p53wt group, 11% (1 of 9) in the MMRd group, and 33% (2 of 6) in the p53abn group.

Details of the patients who recurred are summarized in Table 4. All recurrences were to distant sites, including the peritoneum, lung, and liver. All patients who recurred died following a diagnosis of metastatic disease, with a median overall survival of 36 months (IQR 23.5–50 months). Of the recurrences, two (50%) were p53abn. One of these patients was treated with chemoradiation. The other patient had p53abn serous carcinoma identified in a polyp as well as an MMRp p53wt endometrioid component and was treated with VBT alone. Another patient who recurred had stage IIIA MMRd endometrioid carcinoma and did not receive any adjuvant therapy due to significant comorbidities. The final patient who recurred had a stage IB MMRp p53wt endometrioid carcinoma and received EBRT. This patient recurred 8 months after surgery and at that time was found to have widespread abdominal metastases, which appeared consistent with metastatic endometrial cancer. The patient was later found to have concurrent bilateral invasive lobular carcinoma of the breast. There was no formal tissue diagnosis to confirm whether these metastases originated from endometrium or breast.

### 3.5. Literature Review

Fourteen studies were identified that reviewed the recurrence patterns of endometrial cancer with pelvic lymph node ITCs [11,12,13,14,15,16,17,18,19,20,21,22,23,24]. An overview of all included studies is provided in Table S1. The reported prevalence of ITCs ranged from 1% [15] to 10% [20]. Only two studies reviewed molecular profile [20,23]. In a study by Rios-Doria et al., molecular profile was assessed in 28 patients with ITCs; 14 (50%) were NSMP, 11 (39%) were MMRd, two (7%) were p53abn, and one (3%) was *POLE*mut [20]. Of the seven patients who recurred in this cohort of 125 patients, two were MMRd, one was *POLE*mut, and the remaining four had an unknown molecular profile. The recurrence rate was 18% (2/11) for MMRd and 100% (1/1) for *POLE*mut, with no recurrences in the NSMP or p53abn groups. In another study by Awada et al., molecular profile was only reported for patients with ITCs who recurred [23]. Of three patients with recurrences for which molecular profiling was reported, two were MMRd and one was p53abn.

The type of adjuvant treatment used varied considerably between studies. Chemotherapy, with or without radiation therapy, was the most frequent adjuvant treatment choice for patients with ITCs in eight of fourteen studies [12,16,18,20,21,22,23,24]. Conversely, the majority of patients with ITCs did not receive any adjuvant therapy in four studies [14,15,17,19]. The recurrence rate for endometrial cancer with ITCs was also quite variable, ranging from no recurrences [15,19] up to 33% of patients [12]. In studies that were limited to patients with endometrioid histology, recurrence rates ranged from 5% to 12% [11,20,23,24]. In a study by Cucinella et al. [11], the population was restricted to patients with stage IA endometrioid endometrial carcinoma with ITCs who did not receive adjuvant treatment. In this population, the recurrence rate was 12%.

## 4. Discussion

In this small cohort of patients with endometrial cancer and pelvic lymph node ITCs, recurrence outcomes varied descriptively according to available molecular classification. This suggests that the prognostic significance of molecular subtypes applies to endometrial cancer with ITCs. The detection of ITCs in p53abn endometrial cancer is unlikely to change the management of these malignancies, as they usually will receive chemotherapy with or without EBRT, and despite adjuvant therapy, a substantial portion of p53abn endometrial carcinomas with ITCs recurred with distant metastasis. However, ITCs have the potential to influence treatment decisions in the other molecular subtypes, especially MMRp p53wt cancers with low-risk features. We observed a 4% recurrence rate in MMRp p53wt endometrioid endometrial cancers with pelvic lymph node ITCs. All were negative for *POLE* mutations. Nine (21%) patients were MMRd, six (14%) were p53abn, and 28 (65%) were MMRp p53wt.

Our recurrence rate in the MMRd group was lower than what was reported by Rios-Doria et al. [20] (9% versus 18%, respectively), although both studies were limited by a small MMRd population. In the study by Rios-Doria et al., there were no recurrences among 14 NSMP patients, which appears consistent with our one recurrence of 28 MMRp p53wt patients. Although molecular profile was only assessed in patients with ITCs who recurred in Awada et al. [23], and this population only consisted of three patients, none of the patients who recurred were in the NSMP group and were instead in the MMRd and p53abn groups. These two studies align with our finding that recurrence is uncommon in MMRp p53wt endometrial cancer with ITCs.

The lack of formal treatment guidelines for endometrial cancer with ITCs means that there is great variation in how these cancers are managed. In contrast to similar studies, the majority of MMRp p53wt endometrioid endometrial cancers with ITCs at our center were treated with adjuvant pelvic EBRT alone. None of the papers identified in the literature search reported using EBRT alone as their most common adjuvant therapy option for patients with ITCs. Conversely, the majority of centers opted to treat patients with chemotherapy, with or without EBRT. Although it is difficult to make comparisons given the amount of variability between studies, our overall recurrence rate of 9% is within the recurrence range reported in the literature. Previous studies have suggested no association between adjuvant therapy and recurrence-free survival in endometrial cancer with ITCs [24], which may explain the similar recurrence rates across the literature despite the variability in adjuvant treatment decisions.

The question of whether pelvic lymph node ITCs should change management in endometrial cancer remains unanswered and should remain context-dependent. The aggressive nature of p53abn endometrial cancer is well established, and 50% of the patients who recurred in our study were p53abn. MMRd has been associated with occult lymph node metastases in patients with apparently early-stage endometrial cancer, suggesting that MMRd tumors may have a propensity for lymphatic dissemination that is not always identified by routine surgical staging [25]. Excluding patients in the p53abn or MMRd groups, nearly all patients in our cohort had features that would warrant adjuvant treatment regardless of the presence of ITCs. In total, 86% were stage IB or greater, while 36% had diffuse LVSI. However, pathological features like LVSI should also be considered in context, such as whether they are accompanied by pelvic lymph node ITCs and a MELF pattern of invasion.

The MELF pattern of myoinvasion is a histologic finding in endometrioid endometrial carcinoma of unclear clinical significance that was highly prevalent in our cohort. Nearly all patients with MMRp p53wt endometrial cancer had a MELF pattern of invasion, whereas it was present in 56% of the MMRd group. MELF is associated with lymph node metastases, usually in the form of ITCs [26], as well as diffuse LVSI [27]. Endometrioid endometrial cancer with MELF has been found to harbor an 8.5 times higher risk of developing ITCs [28]. The vast majority of MELF-positive endometrioid carcinomas are NSMP, and such tumors appear to have a low risk of recurrence [27]. This appears consistent with the results of our study, where overall recurrence was 4% in MMRp p53wt endometrioid carcinomas with MELF. However, this observation is confounded by the frequent use of adjuvant treatment, the absence of an ITC-negative comparator group, and the small number of recurrence events. Therefore, a potentially limited metastatic significance of ITCs in the MELF setting remains a hypothesis that requires validation in larger, molecularly characterized cohorts.

A strength of our study is that we have provided an overview of the molecular profile in endometrial cancer with ITCs, of which there are currently limited reports in the literature. We were able to obtain p53 and MMR status in all of our patients. An understanding of molecular profile in these cases will improve risk-stratification for endometrial cancer with ITCs and will help us better understand the clinical significance of ITCs. Re-review of all cases by a single gynecological pathologist also ensured consistency and validity. Furthermore, we provide clinical outcome data for a population of patients with ITCs treated liberally with adjuvant EBRT, of which there are also few reports.

A limitation of this study is that *POLE* testing was unavailable in 70% of our cohort. Thus, complete molecular classification according to the TCGA framework was not possible for the majority of patients. Although tumors with pathogenic *POLE* mutations rarely show nodal metastasis, there may be a limited number of patients in our cohort with ITCs who had *POLE*mut endometrial carcinoma. However, it is highly unlikely that the cases with disease recurrence had *POLE*mut tumors. In a recent study by Bogani et al., only 11 of 164 patients with *POLE*mut tumors had nodal metastases, and there were no recurrences observed after a median follow-up of 7.6 months [29]. *POLE* testing was implemented more recently at our center and is usually only tested in patients where the presence of a pathogenic *POLE* mutation may change management, allowing for treatment de-escalation [30]. As there were no patients with *POLE*mut in our cohort, the impact of ITCs in *POLE*mut endometrial cancer requires further investigation.

The low observed recurrence rate among patients with MMRp p53wt tumors must also be interpreted in the context of treatment. Most patients received adjuvant therapy, and pelvic EBRT was used frequently in this subgroup. Because treatment was not randomized and was determined according to clinicopathological characteristics and multidisciplinary recommendations, the present study cannot distinguish the contribution of tumor biology from that of adjuvant treatment. Importantly, our study did not include a comparator group of patients without ITCs. Therefore, we cannot determine whether ITCs independently influence recurrence risk relative to otherwise comparable patients without nodal tumor cells. The observed outcomes should therefore not be interpreted as establishing the prognostic significance of ITCs themselves. Furthermore, the nearly decade-long study period may have introduced temporal variation in molecular testing, ultrastaging, and adjuvant treatment, while differences in keratin immunohistochemistry use between sentinel and non-sentinel nodes may have influenced ITC detection.

Although we utilized a database of prospectively collected data for patients undergoing endometrial cancer surgery at our center, there is a possibility that not all patients with ITCs were captured by the database. Therefore, the estimation of the prevalence of ITCs at our center is subject to this limitation. To identify patients with ITCs who had not yet been uploaded to the database, additional patients were identified from tumor board documents. This was done to increase our sample size, especially with regard to *POLE* testing, as patients seen more recently were more likely to have testing done. However, this also introduces some selection bias into our cohort. Patients identified from tumor board documents were not included in the calculation for the prevalence of ITCs to account for this bias. Finally, given the significant variability between studies included in the literature review regarding population and adjuvant treatment decisions, comparisons are limited. Prospective trials and randomized controlled trials are required to draw conclusions regarding treatment options for patients with ITCs. Further investigation regarding molecular subtype in ITCs is also warranted, especially with regard to the *POLE*mut subgroup. Increased understanding of the significance of the MELF pattern of invasion in the context of pelvic lymph node ITCs is also required to determine the best treatment for patients with these histopathologic features.

## 5. Conclusions

In this retrospective single-institution cohort of endometrial cancer patients with pelvic lymph node ITCs, recurrence was uncommon among patients with MMRp p53wt tumors, with one of 28 patients experiencing recurrence during a median follow-up of 33 months. However, the small cohort, limited number of recurrence events, incomplete *POLE* testing, absence of an ITC-negative comparator, and frequent use of adjuvant treatment limit interpretation of these findings. Larger, molecularly complete cohorts with appropriate comparator groups and longer follow-up are needed to clarify the independent prognostic significance of ITCs and the potential relationship between MELF, molecular subtype, and recurrence.

## Figures and Tables

**Table 1:** Pathologic characteristics of isolated tumor cells (*n* = 43). FIGO, Federation of Gynecology and Obstetrics; ITCs, isolated tumor cells; MELF, microcystic, elongated, and fragmented pattern of invasion; LVSI, lymphovascular space invasion.

		*n* (%)
Histology	Endometrioid carcinoma	39 (90.7%)
	Serous carcinoma	3 (7%)
	Mixed carcinoma	1 (2.3%)
FIGO grade	1	24 (55.8%)
	2	12 (27.9%)
	3	4 (9.3%)
	High-grade	3 (7%)
pT	pT1a	11 (25.6%)
	pT1b	20 (46.5%)
	pT2	10 (23.3%)
	pT3a	1 (2.3%)
	pT3b	1 (2.3%)
FIGO 2023 stage	IA	11 (25.6%)
	IB	20 (46.5%)
	II	10 (23.3%)
	IIIA	1 (2.3%)
	IIIB	1 (2.3%)
MMR	Normal	34 (79.1%)
	Abnormal	9 (20.9%)
p53	Wild-type	37 (86%)
	Abnormal	6 (14%)
*POLE*	Negative	13 (30.2%)
	Unknown	30 (69.8%)
Myometrial invasion	Not identified	1 (2.3%)
	<50%	10 (23.3%)
	>50%	31 (72.1%)
	Unknown	1 (2.3%)
Cervical stromal invasion	Not identified	33 (76.7%)
	Present	10 (23.3%)
MELF pattern of invasion	Identified	35 (81.4%)
	Not identified	5 (11.6%)
	N/A	3 (7%)
LVSI	Absent	15 (34.9%)
	Focal	9 (20.9%)
	Diffuse	12 (27.9%)
	Present, not specified	7 (16.3%)
Number of nodes with ITCs	1	36 (83.7%)
	2 or more	7 (16.3%)
Laterality of ITCs	Left	20 (46.5%)
	Right	16 (37.2%)
	Bilateral	7 (16.3%)

**Table 2:** Pathologic characteristics of MMR-proficient p53 wild-type endometrial cancer with isolated tumor cells (*n* = 28). FIGO, Federation of Gynecology and Obstetrics; ITCs, isolated tumor cells; LVSI, lymphovascular space invasion; MELF, microcystic, elongated, and fragmented pattern of invasion.

		*n* (%)
Histology	Endometrioid carcinoma	28 (100%)
FIGO grade	1	19 (67.9%)
	2	7 (25%)
	3	2 (7.1%)
pT	pT1a	4 (14.2%)
	pT1b	15 (53.5%)
	pT2	8 (28.8%)
	pT3b	1 (3.5%)
FIGO 2023 stage	IA	4 (14.2%)
	IB	15 (53.5%)
	II	8 (28.8%)
	IIIB	1 (3.5%)
*POLE*	Negative	9 (32.1%)
	Unknown	19 (67.9%)
Myometrial invasion	<50%	4 (14.3%)
	>50%	24 (85.7%)
Cervical stromal invasion	Not identified	20 (71.4%)
	Present	8 (28.6%)
MELF pattern of invasion	Identified	27 (96.4%)
	Not identified	1 (3.6%)
LVSI	Absent	7 (25%)
	Focal	5 (17.9%)
	Diffuse	10 (35.7%)
	Present, not specified	6 (21.4%)
Number of nodes with ITCs	1	22 (78.6%)
	2 or more	6 (21.4%)
Laterality of ITCs	Left	12 (42.9%)
	Right	10 (35.7%)
	Bilateral	6 (21.4%)

**Table 3:** Adjuvant treatment decisions for endometrial cancer with isolated tumor cells by molecular profile. Chemo, chemotherapy; EBRT, external beam radiation therapy; MMRd, mismatch-repair-deficient; MMRp, mismatch-repair-proficient; p53abn, p53-abnormal; p53wt, p53 wild-type; VBT, vaginal brachytherapy.

	MMRp p53wt (*n* = 28)	MMRd (*n* = 9)	p53abn (*n* = 6)	Overall (*n* = 43)
EBRT alone	12	2	0	14 (32.6%)
EBRT + VBT	4	1	0	5 (11.6%)
EBRT + Chemo	0	0	2	2 (4.7%)
EBRT + Chemo + VBT	2	0	1	3 (7%)
Chemo alone	1	0	1	2 (4.7%)
Chemo + VBT	0	0	1	1 (2.3%)
VBT alone	3	1	1	5 (11.6%)
No adjuvant treatment	6	5	0	11 (25.6%)

**Table 4:** Characteristics of endometrial cancer patients with ITCs who experienced a recurrence. Chemo, chemotherapy; EBRT, external beam radiation therapy; ITCs, isolated tumor cells; LVSI, lymphovascular space invasion; MELF, microcystic, elongated, and fragmented pattern of invasion; MMRd, MMR-deficient; MMRp, MMR-proficient; OS, overall survival; p53abn, p53-abnormal; p53wt, p53 wild-type; RFS, recurrence-free survival; VBT, vaginal brachytherapy.

Age	Grade	Histology	Stage	Molecular	MELF	LVSI	Lymph Nodes with ITCs	Adjuvant Treatment	Area of Recurrence	RFS (Months)	OS (Months)
76	High-grade	Serous	IB	p53abn	N/A	Not identified	1/14	Chemo + EBRT	Distant	44	61
80	1	Endometrioid	IIIA	MMRd	No	Diffuse	1/2	None	Distant	18	33
83	2	Endometrioid	IB	MMRp p53wt	Yes	Present, not specified	1/3	EBRT	Distant	8	39
81	2	Mixed endometrioid and serous	IB	p53abn in serous component, MMRp p53wt in endometrioid component	Yes	Diffuse	1/4	VBT	Distant	8	14

## Data Availability

The data presented in this study are available on request from the corresponding author due to patient confidentiality.

## Supplementary Materials

The following supporting information can be downloaded at: https://www.mdpi.com/article/10.3390/cancers18203293/s1, Table S1: Characteristics of studies reviewing recurrence in endometrial cancer with pelvic lymph node isolated tumor cells. B, brachytherapy; C, chemotherapy; ITCs, isolated tumor cells; Lap, laparoscopic; MMRd, MMR-deficient; n, population; NA, not assessed; NSMP, no specific molecular profile; O, open; p53abn, p53 abnormal; POLEmut, POLE mutated; R, robot-assisted; RT, external beam radiation therapy; SLN, sentinel lymph node; USA, United States of America; V, vaginal.
