# Supplementary material for: Clinicopathologic Characteristics, Molecular Profile, Treatment, and Recurrence in Endometrial Cancer with Pelvic Lymph Node Isolated Tumor Cells
Source: Cancers (Basel). 2026 Oct 13;18(20):3293. doi: 10.3390/cancers18203293 (PMC13702782; doi:10.3390/cancers18203293)
Supplement: Supplementary file 1 [file cancers-18-03293-s001.zip › cancers-4598359-supplementary.pdf]

cancers

Table S1. Characteristics of studies reviewing recurrence in endometrial cancer with pelvic lymph node isolated tumor cells. B, brachytherapy; C, chemotherapy; ITCs, isolated tumor cells; Lap, laparoscopic; MMRd, MMR-deficient; n, population; NA, not assessed; NSMP, no specific molecular profile; O, open; p53abn, p53 abnormal; POLEmut, POLE mutated; R, robot-assisted; RT, external beam radiation therapy; SLN, sentinel lymph node; USA, United States of America; V, vaginal.

Study	Country	Study design	Population	Total n	n with ITCs	Population prevalence of ITCs (%)	Surgery approach	% endometrioid histology	Molecular profile	Adjuvant treatment	% with recurrence	Median follow-up time (months)
St. Clair, 2016 [1]	USA	Retrospective single-center cohort	Endometrial cancer with SLN biopsy	844	23	2.7	R (52.5%) Lap (30.4%) O (17.1%)	82.6	NA	C (87%) RT (8.7%) None (4.3%)	8.7	26
Plante, 2017 [2]	Canada	Retrospective single-center cohort	Endometrial cancer with SLN biopsy	519	31	6	O (47%) Lap (33%) R (20%)	90.3	NA	C +/- RT (35%) RT (32%) None/B (32%)	3.2	29
Aloisi, 2018 [3]	USA	Retrospective single-center cohort	Endometrial cancer with positive pelvic lymph nodes	207	71	NA	R (55.6%) O (28%) Lap (16.4%)	64	NA	B +/- RT (90%)	16.9	32
Backes, 2019 [4]	USA	Prospective single-center clinical trial	Endometrial cancer with SLN biopsy	204	9	4.4	R	88.9	NA	None (55.6%) B (22.2%) C (11.1%) RT (11.1%)	0	31
Garcia Pineda, 2020 [5]	Spain	Retrospective single-center cohort	Endometrial cancer with SLN biopsy	230	6	2.6	Lap (88.4%) O (10.1%) V (1.5%)	83.3	NA	C +/- RT (50%) None (33.3%) RT +/- B (16.7%)	33.3	60
Goebel, 2020 [6]	USA	Retrospective single-center cohort	Endometrial cancer with SLNs originally reported as negative	155	21	NA	Lap	95.2	NA	None (62%) C + RT (19%) B (19%)	0	31.5
Backes, 2021 [7]	International	Retrospective multi-center cohort	Stage I/II endometrioid endometrial carcinoma with SLN ITCs	175	175	NA	Not specified	100	NA	C +/- RT (45%) None/B (43%) RT (12%)	5.1	31
Ghoniem, 2021 [8]	International	Retrospective multi-center cohort	Endometrial cancer with ITCs or micrometastasis	247	132	NA	Not specified	87.9	NA	C and/or RT +/- B (62.9%) None/B (37.1%)	12.8	29.6
Buda, 2023 [9]	International	Retrospective multi-center cohort	Endometrial cancer with SLN biopsy	1428	26	1.8	R/Lap (89.4%) O (10.1%) R/Lap + O (0.5%)	73.1	NA	C + RT (46.2%) None (26.9%) RT (19.2%) C (7.7%)	19.2	33.3
Lavecchia, 2023 [10]	International	Retrospective multi-center cohort	Endometrial cancer with SLN biopsy	1012	12	1.2	R (75%) O (16.7%) Lap (8.3%)	91.7	NA	None (41.7%) C + RT (16.7%) C + RT + B (16.7%) RT (8.3%) B (8.3%)	0	27

										C (8.3%)		
Cucinella, 2024 [11]	International	Retrospective multicenter cohort	Stage IA grade 1 or 2 endometrioid endometrial carcinoma with ITCs who did not receive adjuvant therapy, with node-negative control group	494	42	NA	Not specified	100	NA	None	11.9	27.6
Dinoi, 2024 [12]	USA	Retrospective single-center cohort	Endometrial cancer with positive pelvic lymph nodes	103	36	NA	R (88.3%) Lap (7.8%) O (0.4%)	94.4	NA	None/B (38.9%) C + RT (25%) RT +/- B (13.9%) C +/- B (13.9%)	11.1	30
Rios-Doria, 2024 [13]	USA	Retrospective single-center cohort	Stage IA, IB, or II endometrioid endometrial cancer with SLN biopsy	1214	125	10	R/Lap (89%) O (11%)	100	NSMP (14/28, 50%) MMRd (11/28, 39.3%) p53abn (2/28, 7.1%) POLEmut (1/28, 3/6%)	C +/- RT (62.4%) RT (24.8%) None (12.8%)	5.6	36.5
Awada, 2025 [14]	USA	Retrospective single-center cohort	Early-stage high-intermediate risk* endometrioid endometrial cancer treated with C + RT or RT	1480	48	NA	R (97.9%) O (2.1%)	100	MMRd (2/3, 66.7%) p53abn (1/3, 33.3%)**	C + RT (67%) RT (31%) B (2%)	8.3	63.2 (C + RT cohort) 28 (RT or B cohort)

*defined as age ≥70 with ≥1 risk factor, age ≥50 with ≥2 risk factors, or age <50 with 3 risk factors, with risk factors including FIGO stage I/II outer third depth of invasion, grade 2-3 histology, and positive lymphovascular space invasion.**molecular profile only reported for patients with recurrences.

References

1. C.M. St Clair, A.G.Z. Eriksson, J.A. Ducie, E.L. Jewell, K.M. Alektiar, M.L. Hensley, R.A. Soslow, N.R. Abu-Rustum, M.M.J. Leitao, Low-Volume Lymph Node Metastasis Discovered During Sentinel Lymph Node Mapping for Endometrial Carcinoma., Ann Surg Oncol 23 (2016) 1653–9. https://doi.org/10.1245/s10434-015-5040-z.
2. M. Plante, J. Stanleigh, M.-C. Renaud, A. Sebastianelli, K. Grondin, J. Gregoire, Isolated tumor cells identified by sentinel lymph node mapping in endometrial cancer: Does adjuvant treatment matter?., Gynecol Oncol 146 (2017) 240–246. https://doi.org/10.1016/j.ygyno.2017.05.024.
3. A. Aloisi, J.M. Casanova, J.H. Tseng, K.A. Seader, N.T. Nguyen, K.M. Alektiar, V. Makker, S. Chiang, R.A. Soslow, M.M.J. Leitao, N.R. Abu-Rustum, Patterns of FIRST recurrence of stage IIIC1 endometrial cancer with no PARAAORTIC nodal assessment., Gynecol Oncol 151 (2018) 395–400. https://doi.org/10.1016/j.ygyno.2018.09.021.
4. F.J. Backes, D. Cohen, R. Salani, D.E. Cohn, D.M. O'Malley, E. Fanning, A.A. Suarez, J.M. Fowler, Prospective clinical trial of robotic sentinel lymph node assessment with isosulfane blue (ISB) and indocyanine green (ICG) in endometrial cancer and the impact of ultrastaging (NCT01818739)., Gynecol Oncol 153 (2019) 496–499. https://doi.org/10.1016/j.ygyno.2019.03.252.

5. V. Garcia Pineda, A. Hernandez Gutierrez, M. Gracia Segovia, J. Siegrist Ridruejo, M.D. Diestro Tejeda, I. Zapardiel, Low-Volume Nodal Metastasis in Endometrial Cancer: Risk Factors and Prognostic Significance., J. Clin. Med. 9 (2020). https://doi.org/10.3390/jcm9061999.
6. E.A. Goebel, J.D. St Laurent, M.R. Nucci, C.M. Feltmate, Retrospective detection of isolated tumor cells by immunohistochemistry in sentinel lymph node biopsy performed for endometrial carcinoma: is there clinical significance?., Int J Gynecol Cancer 30 (2020) 291–298. https://doi.org/10.1136/ijgc-2019-000934.
7. F.J. Backes, A.S. Felix, M. Plante, J. Grégoire, S.A. Sullivan, E.C. Rossi, E.J. Tanner, K.I. Stewart, P.T. Soliman, R.W. Holloway, N.R. Abu-Rustum, M.M. Leitao, Sentinel lymph node (SLN) isolated tumor cells (ITCs) in otherwise stage I/II endometrioid endometrial cancer: To treat or not to treat?, Gynecol Oncol 161 (2021) 347–352. https://doi.org/10.1016/j.ygyno.2021.02.017.
8. K. Ghoniem, A.M. Larish, G. Dinoi, X.C. Zhou, M. Alhilli, S. Wallace, C. Wohlmuth, G. Baiocchi, N. Tokgozoglu, F. Raspagliesi, A. Buda, V. Zanagnolo, I. Zapardiel, N. Jagasia, R. 2nd Giuntoli, A. Glickman, M. Peiretti, M. Lanner, E. Chacon, J. Di Guilmi, A. Pereira, E. Laas, A. Fishman, C.C. Nitschmann, S. Parker, A. Joehlin-Price, B. Lees, A. Covens, L. De Brot, C. Taskiran, G. Bogani, C. Paniga, F. Multinu, A. Hernandez-Gutierrez, A.L. Weaver, M.E. McGree, A. Mariani, Oncologic outcomes of endometrial cancer in patients with low-volume metastasis in the sentinel lymph nodes: An international multi-institutional study., Gynecol Oncol 162 (2021) 590–598. https://doi.org/10.1016/j.ygyno.2021.06.031.
9. A. Buda, C. Paniga, S. Taskin, M. Mueller, I. Zapardiel, F. Fanfani, A. Puppo, J. Casarin, A. Papadia, E. De Ponti, T. Grassi, J. Mauro, H. Turan, D. Vatansever, M. Gungor, F. Ortag, S. Imboden, V. Garcia-Pineda, S. Mohr, F. Siegenthaler, S. Perotto, F. Landoni, F. Ghezzi, G. Scambia, C. Taskiran, R. Fruscio, The Risk of Recurrence in Endometrial Cancer Patients with Low-Volume Metastasis in the Sentinel Lymph Nodes: A Retrospective Multi-Institutional Study., Cancers (Basel) 15 (2023). https://doi.org/10.3390/cancers15072052.
10. M. Lavecchia, J.-H. Jang, H.-J. Lee, S. Pin, H. Steed, J.-Y. Lee, S. Ghosh, J.S. Kwon, Sentinel lymph node biopsy in endometrial cancer: The new norm - A multicentre, international experience., Surg Oncol 48 (2023) 101922. https://doi.org/10.1016/j.suronc.2023.101922.
11. G. Cucinella, G. Schivardi, X.C. Zhou, M. AlHilli, S. Wallace, C. Wohlmuth, G. Baiocchi, N. Tokgozoglu, F. Raspagliesi, A. Buda, V. Zanagnolo, I. Zapardiel, N. Jagasia, R. Giuntoli, A. Glickman, M. Peiretti, M. Lanner, E. Chacon, J. Di Guilmi, A. Pereira, E. Laas-Faron, A. Fishman, C.C. Nitschmann, K. Kurnit, K. Moriarty, A. Joehlin-Price, B. Lees, A. Covens, L. De Brot, C. Taskiran, G. Bogani, F. Landoni, T. Grassi, C. Paniga, F. Multinu, L.A. De Vitis, A. Hernandez, S. Mastroyannis, K. Ghoniem, V. Chiantera, M. Shahi, A.J. Fought, M. McGree, A. Mariani, G. Glaser, Prognostic value of isolated tumor cells in sentinel lymph nodes in low risk endometrial cancer: results from an international multi-institutional study., Int J Gynecol Cancer 34 (2024) 179–187. https://doi.org/10.1136/ijgc-2023-005032.
12. G. Dinoi, K. Ghoniem, Y. Huang, V. Zanfagnin, G. Cucinella, C. Langstraat, G. Glaser, A. Kumar, A. Weaver, M. McGree, F. Fanfani, G. Scambia, A. Mariani, Endometrial cancer with positive sentinel lymph nodes: pathologic characteristics of metastases as predictors of extent of lymphatic dissemination and prognosis., Int J Gynecol Cancer (2024). https://doi.org/10.1136/ijgc-2023-005181.
13. E. Rios-Doria, N.R. Abu-Rustum, K.M. Alektiar, V. Makker, Y.L. Liu, D. Zamarin, C.F. Friedman, C. Aghajanian, L.H. Ellenson, S. Chiang, B. Weigelt, J.J. Mueller, M.M. Leitao, Prognosis of isolated tumor cells and use of molecular classification in early stage endometrioid endometrial cancer., Int J Gynecol Cancer (2024). https://doi.org/10.1136/ijgc-2024-005522.
14. A. Awada, F.O. Recio, T.M. Kuhn, S. Ahmad, J. Zhu, N.D. McKenzie, J.E. Kendrick, R.W. Holloway, Recurrence and survival in high-intermediate risk endometrial cancers with isolated tumor cell lymph node metastasis., Gynecol Oncol Rep 57 (2025) 101684. https://doi.org/10.1016/j.gore.2025.101684.
